# Nonvolatile and tunable switching of lateral photo-voltage triggered by laser and electric pulse in metal dusted metal-oxide-semiconductor structures

**DOI:** 10.1038/srep32015

**Published:** 2016-08-18

**Authors:** Peiqi Zhou, Zhikai Gan, Xu Huang, Chunlian Mei, Meizhen Huang, Yuxing Xia, Hui Wang

**Affiliations:** 1 The State Key Laboratory of Advanced Optical Communication Systems and Networks, Department of Physics and Astronomy, the Key Laboratory of Thin Film and Nano-microfabrication Technology of the Ministry of Education, and Department of Instrument Science and Engineering, Shanghai JiaoTong University, 800 Dongchuan Rd, Shanghai 200240, P. R. China

## Abstract

Owing to the innate stabilization of built-in potential in p–n junction or metal-oxide-semiconductor structure, the sensitivity and linearity of most lateral photovoltaic effect (LPE) devices is always fixed after fabrication. Here we report a nonvolatile and tunable switching effect of lateral photo-voltage (LPV) in Cu dusted ultrathin metal-oxide-semiconductor structure. With the stimulation of electric pulse and local illumination, the sensitivity and linearity of LPV can be adjusted up and down in a nonvolatile manner. This phenomenon is attributed to a controllable change of the Schottky barrier formed between the metal layer and silicon substrate, including the consequent change of film resistivity. This work may widely improve the performance of existing LPE-based devices and suggest new applications for LPE in other areas.

Since the lateral photovoltaic effect (LPE) was first discovered by Schottky in ref. 1 and explicitly raised by Wallmark in ref. 2, its unique features (LPV varies linearly with irradiation position with high sensitivity) have given birth to extensive applications in many fields, which can be divided into two categories. In one category, LPE was applied to design new optical transducers and sensors, and a prominent example is position-sensitive detectors^3^,^4^,^5^ (PSD), which is widely used in various experimental measurements^6^,^7^,^8^ (especially in high energy physics experiments). In the other category, LPE is used as a method to explore new physical phenomena^9^,^10^,^11^ or measure other physical quantities^12^,^13^ such as sheet resistivity, junction conductance, and built-in potential. Because of these application values and physical properties, over the past few decades, a fair amount of work has been done to improve the sensitivity and linearity of LPE in varieties of PN junction type or MOS type structures^14^,^15^,^16^,^17^,^18^, such as interface modification^19^, thickness control^20^, quantum dots embedding^21^, ions implantation^22^, external bias^23^,^24^,^25^, and so on ref. 26,27. However, almost all these methods are either applied in the fabrication stage or invalid without continuous voltage input. In other words, nonvolatile optimization measures of LPV under working condition have scarcely been reported. In this paper, we investigated the LPE in Cu (dusted)-SiO_2_-Si structure, differing from previous studies, the sensitivity and linearity of LPV, can be freely adjusted by an electric pulse with assist of light after fabrication. We attribute this adjustment to the control of Schottky barrier height (SBH) between Cu and Si layer. This discovery may widely improve the performance of existing LPE-based devices. Moreover, it may immensely expand the application space of LPE.

## Methods

**Figure 1:**
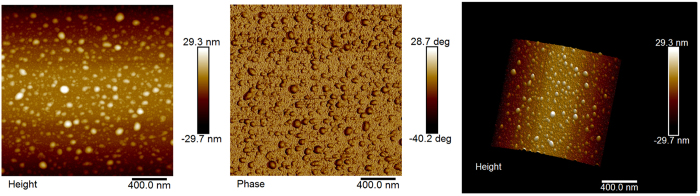
The height image, phase image and 3D image of 2.46 nm thickness sample. Showing the metal film is actually discontinuous nanoparticles.

We deposited Cu films on n-type Si (1 1 1) (0.3 mm with an oxide layer) at room temperature by radio-frequency sputtering. The base pressure of the vacuum system prior to deposition was 4.1 × 10–4 Pa, and the argon gas pressure was 0.7 Pa during the deposition. The deposition rates, determined by stylus profile meter on thick calibration samples, were 0.41 Å/s. A semiconductor laser (415 nm wavelength) was used to scan the sample, with a 50 μm laser spot diameter and a 3 mW laser power reaching the sample. All the contacts (less than 1 mm in diameter) to the films were formed by alloying indium and showed no measurable rectifying behavior (ohmic contact). Several ultrathin Cu films were deposited with various thicknesses from 0.4 nm to 4 nm. Figure 1 shows the AFM images of one sample, which indicates that the super thin Cu films are discontinuous, actually nanoparticles in light of the first stage are of Volmer-Weber growth mode. More direct visualized SEM and AFM images of this discontinuity and the growth mode can be found in other previous reports^28^,^29^. Therefore, the deposited Cu thickness in our samples is only a nominal thickness.

## Results and Discussion

**Figure 2:**
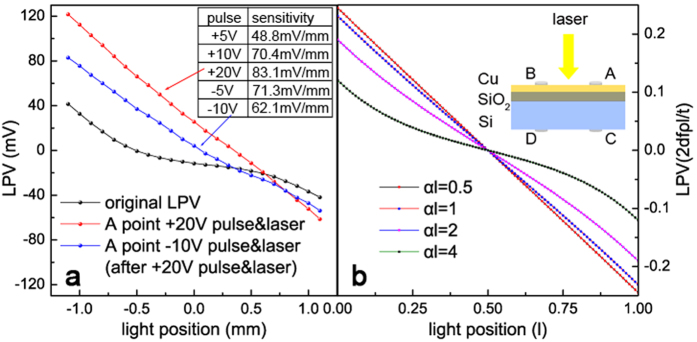
(**a**) The response of LPV to the pulse & laser stimulation. Black line: original LPV curve without stimulation. Red line: LPV curve after a +20 V pulse & laser stimulation at A point. Blue line: LPV curve after a −10 V pulse & laser stimulation at A point on the basis of the blue line. Table: the variation of LPV sensitivity with the change of pulse voltage in sequence. (**b**) The evolution of LPV with the increase of *αl* based on the current continuity equation and Ohm’s low.

The original LPV (photo-voltage between A point and B point, see the illustration in Fig. 2(b)) curve of our sample was shown in Fig. 2(a) in black line. We can see the sensitivity of LPV is only about 38 mV/mm, and the linearity is not very ideal either. Then, we applied a +5 V voltage pulse (“+” means the anode is located on the Cu side) on A point, which was irradiated by the laser. Once again, we measured the LPV curve of this sample, the sensitivity increased to 48.8 mV/mm. And it further increased to 70.4 mV/mm after a +10 V pulse, until reaching 83.1 mV/mm after a +20 V pulse, more than double the original state, as shown in red line. At the same time, the linearity of the curve is also much better than the original state, too. To investigate the stability of this improvement, we continuously measured the LPV for a week and didn’t find obviously change. This is a very exciting result. It means the voltage pulse permanently changed the internal structure of this sample in some degree. To further investigate this phenomenon, we reversed the polarity of pulse, applied a −5 V pulse on A point (still with the assist of laser on A point), consequently, the sensitivity dropped to 71.3 mV/mm. We increased the voltage to −10 V, and then the sensitivity dropped to 62.1 mV/mm, with the LPV curve shown in the blue line. We can see the sensitivity and linearity both reduced obviously after the pulse was reversed, which indicating this pulse & laser adjustment is reversible. In other words, with the help of local illumination, we can use different pulses enhance or reduce the performance of LPV. In order to confirm the necessity of laser illumination on pulse point, we repeated this experiment without laser on the control group, which is a sample tailored from a same original one as in the case of the experimental group. Interestingly, without the assist of laser, the change of sensitivity turns to a negligible level, which is not shown in the figure. This result indicates that this tunable LPV effect is stimulated by voltage pulse combined with illumination, and both of these two factors are indispensable. Therefore, we have reasons to infer that the photon-generated carriers play an important role in this effect.

To account for this phenomenon, we start with the conventional derivation of LPV in MOS structure. Based on the current continuity equation and Ohm’s low, we can elicit the electric potential of every point on Cu surface, and then the LPV between electrodes A and B. The derivation is as follow^26^,^30^:


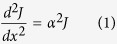



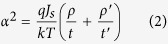



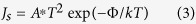



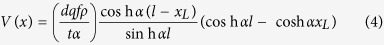



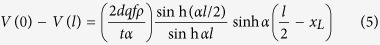



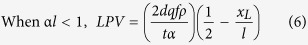


where *α* is the spatial decay parameter, *J*_*s*_ is the Schottky current, *ρ* is the metal film resistivity, *ρ*′ is the semiconductor film resistivity, *t* the metal film thickness, *t*′ the semiconductor film thickness, A* is Richardson constant, Φ is Schottky barrier height, *d* is the diameter of the light spot, *f* is the light generated carrier flux which is collected, *l* is the distance between two electrodes, and *x*_*L*_ is the position of the light spot.

From equation (5), we can see the sensitivity of LPV is proportional to *ρ/αt*, and the linearity mainly relies on the spatial decay parameter *α* and *l*. Figure 2(b) shows the evolution of LPV with the increase of *αl*, when *αl* ≤ 1, the curve shows good linearity, but when *αl* > 1, linearity and sensitivity drop quickly with the increase of *αl*. In practical applications, *l* usually relates to the device measurement range, and it should not be sacrificed to improve linearity. So the parameter *α*, which is positive correlation with *ρ* and negative correlation with Φ and *t*, is the key factor in linearity. Therefore, if we want to improve the performance (both sensitivity and linearity) of LPV devices, we must try to increase the metal film resistivity *ρ* and the Schottky barrier height Φ, and reduce the thickness of metal film *t* at the same time. However, *ρ* is a function strongly depended on *t*, and the Schottky barrier, as is well known, is determined by the metal work function, the electron affinity of the semiconductor, the degree of Fermi-level pinning S = d*ϕ*_*B*_/d*ϕ*_*M*_^31^, and the metal coverage for ultrathin film^32^. Namely, after device fabrication (*t* is settled), *ρ* and Φ are almost fixed, so that most of the previous attempts to optimize LPV are in the stage of fabrication. The most striking feature of our study is that our experiment operations, light-assisted electric pulse, changed the metal film resistivity and the Schottky barrier height when the thickness of metal film is already invariable.

**Figure 3:**
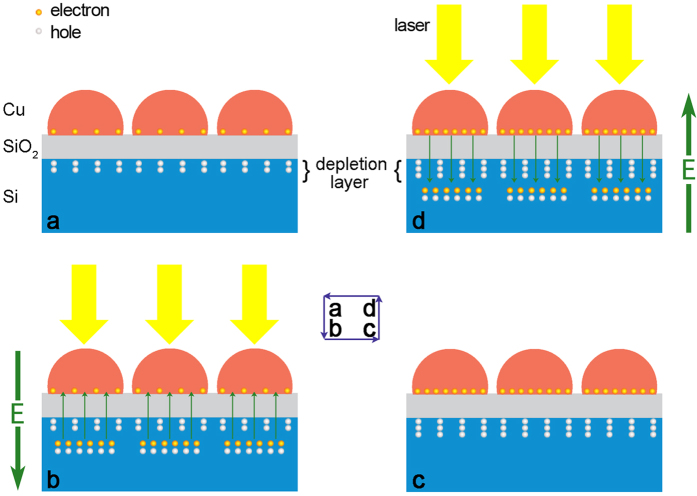
Schematic of the charge states under a pair of positive and negative pulses. (**a**) Original state with low built-in electric field and thin depletion. (**b**) During the positive pulse, the generated electrons are moving from Si to Cu. (**c**) Excited state with high built-in electric field and thick depletion. After the pulse, the generated electrons trapped on the Cu particles. (**d**) During the negative pulse, the trapped electrons are being released to the Si layer.

**Figure 4:**
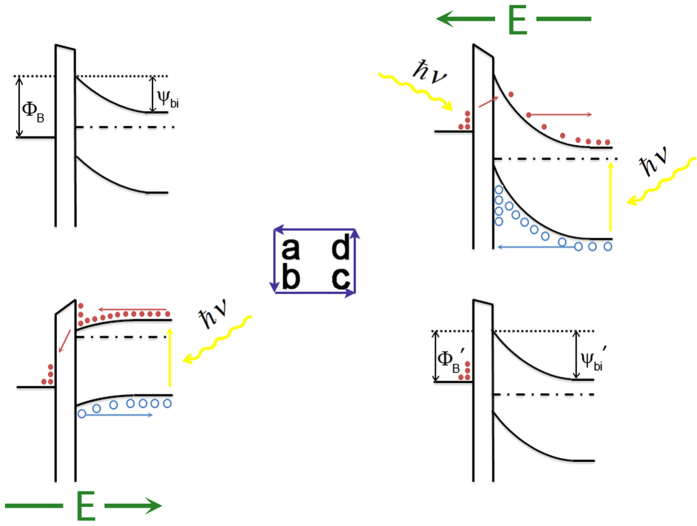
Band diagrams of the charge states under a pair of positive and negative pulses. (**a**) Original state. (**b**) During the positive pulse, the generated electrons are moving from Si to Cu. (**c**) Excited state: after the pulse, the generated electrons trapped on the Cu particles, reducing the barrier on the side of metal and enhancing the built-in electric field. (**d**) During the negative pulse, the trapped electrons are being released to the Si layer.

A model concerning the trapping of generated carriers is suggested to account for the variation of SBH. Firstly, it is worth mentioning that our sample is different from conventional MOS structures. The metal layer in our sample is so thin (less than 2 nm) that actually it is nanoparticles rather than bulk metal or continuous film due to the first stage of the Volmer-Weber growth mode. This discontinuity results in a very low surface conductivity^33^,^34^, on account of the difficult transport between adjacent metal particles for electrons with low energy. Hence, these particles can be treated as potential wells for electrons in the interface. Additionally, due to the presence of oxide layer between Cu and Si, once electrons from Si layer are trapped in these potential wells^35^,^36^, the oxide layer will act as a barrier and prevent these electrons from returning to the Si film. These features of structure give the SBH tunable character. When a laser (the photon energy is greater than the energy gap of Si) is irradiating at a certain point on the sample, a current will flow from Si to Cu (electrons migration from Cu to Si). Meanwhile, if a positive voltage is applied at the same point, some light-generated electrons will drift from the Si layer to the Cu layer under the drive of this external voltage. So the ultima flow direction of the generated electrons is the result of the game between the applied voltage and the build-in internal electric field. If the voltage is high enough, a fraction of the total number of electrons will tunnel through the oxide layer, and then be trapped in potential wells at the surface. Even, therefore, after the voltage is removed, some light-generated electrons still stay at the surface due to the block effect of the oxide layer^37^,^38^. Because of these trapped electrons, the charge distribution in the semiconductor below the interface will be altered, which can be dealt with as if it is an image-charge forming a dipole layer with the electrons trapped in the wells. This charge readjustment leads to a larger build-in internal electric field, i.e. the increase of SBH^39^, thereupon, will increase the sensitivity and linearity of LPV. It is worth noting that the trapped electrons will produce a feeble lateral electric field, which generates the translation of LPV zero point, just as the red line in Fig. 2(a). Conversely, if a negative pulse & laser is subsequently applied in the same position, the trapped electrons will be released and flow back to the Si layer, neutralizing the positive space charge, and consequently reducing the SBH towards its former level. Moreover, based on this model it is easy to understand why the assist of laser illumination is necessary, as it provides a large number of photo-generated electrons, thereby allowing sufficient electrons to be trapped in the surface potential wells. A schematic illustration and band diagrams of these changes in charge states, following the application of a pair of positive and negative pulses, is given in Figs 3 and 4.

There are some indispensable explanations for the difference of Fermi level in Fig. 4(a,c). This difference is caused by the unique structure of our sample. During the formation of Schottky barrier, the oxide layer is transparent for the high energy electrons on side of semiconductor and insulated for the low energy electrons on side of metal. With the loss of electrons, the Fermi level of semiconductor drops until the oxide is insulated for the electrons in semiconductor, too. Therefore, the existence of an oxide layer may afford a difference of Fermi level and provide charge trapping space for electrons. As a result, we need to separately discuss the barrier on the side of metal Φ_B_ and the built-in electric potential ψ_bi_, which always keep a constant difference value E_n_ = E_C_−E_F_ in conventional Schottky contact. After a positive pulse & laser treatment, some generated electrons are trapped in the metal particle. These electrons raise the Fermi level of metal particle^32^, which leads to a lower Φ_B_. At the same time, the increased holes in depletion region will enhance the built-in potential ψ_bi_. Therefore, the difference between Φ_B_ and ψ_bi_ isn’t constant any more.

**Figure 5:**
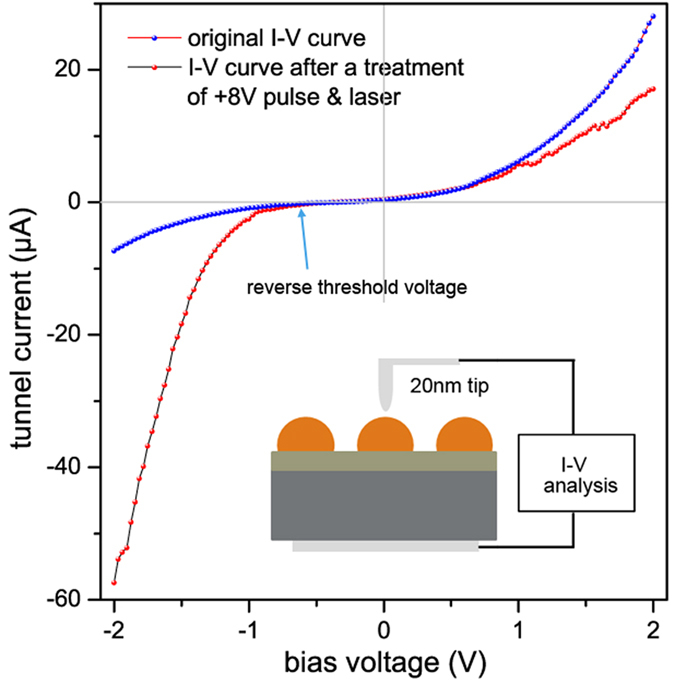
The schematic diagram and I-V curve of single particle through FastScan-AFM.

The I-V characteristic curve shown in Fig. 5 is helpful to verify the unidirectional tunnel property of oxide layer and change of Φ_B_ and ψ_bi_. This curve measured by Fastscan-AFM with a 20 nm tip, and the testing schematic is shown in Fig. 5. As we can see, there is a threshold voltage in the reverse region, indicating that electrons can’t tunnel oxide layer under this voltage, so the oxide layer is insulated for the electron on the metal side without bias. After a +8 V pulse & laser treatment, the reverse current increases notably and the forward current drops. This result is consisted with the change of Φ_B_ and ψ_bi_. Because Φ_B_ mainly influences electrons moving from metal to semiconductor, and ψ_bi_, on the contrary, prevent electrons moving from semiconductor to metal. Here it is necessary to clarify that, the Schottky current *J*_*s*_in equation (3) is the forward current without bias, therefore, Φ in equation (4) is equal to ψ_bi_ + E_n_, which is the SBH we discussed before.

**Figure 6:**
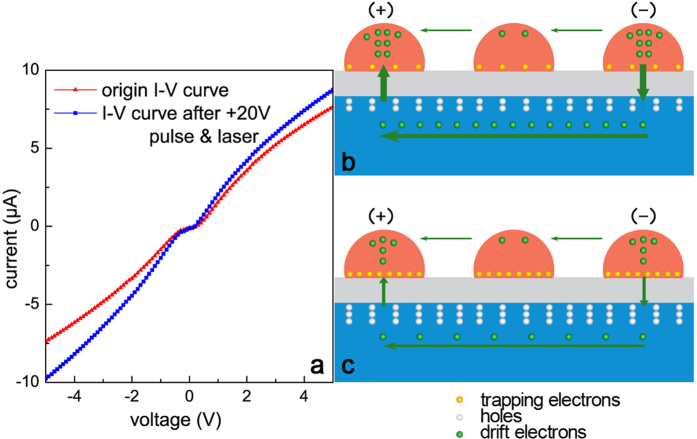
The increase of metal film resistivity caused by the increase of SBH. (**a**) The transformation of I-V curve between point A and B before and after a +20V pulse & laser stimulation. (**b,c**) Schematic of the electroconduct of the Cu ultrathin film, including electrons hopping between Cu islands and electrons detouring through the Si layer.

However, the increase of sensitivity of LPV in our experiment is as large as 120%, which can not be merely explained by the decrease of *α*. During the measurements, another critical factor was found: the metal film resistivity *ρ* increased over 30% after the treatment of +20 V pulse & laser (see Fig. 6). In our model, this change is associated with the increase of SBH. As shown in Fig. 6, the electroconductibility of the Cu ultrathin film mainly comes from two parts: one is electrons hopping from one island to another, the other is electrons detouring through the Si layer. For our sample, the Cu film is thinner than 10 nm and the oxide layer is not very thick, which results in the hopping probability is very low^33^,^34^ and the detouring is relatively easy for electrons so that the second path of electron conduction occupy a dominant position. For this part, the SBH play a role of preventing electrons from tunneling the interface layer, therefore, the increase of SBH will increase the metal film resistivity. Figure 6 shows the transformation of I-V curve between point A and B before and after a +20 V pulse & laser, and the reduction of slope signifies the increase of resistivity. Certainly, this effect only becomes noticeable when the metal film is thin enough that the metal islands have not begun to grow and coalesce.

The effect of another two materials Ti and Pt have also been investigated, whose work function is respective lower and higher than Cu. For the same thickness samples, the improvement of LPV on A point under a same +20 V pulse & laser treatment is shown in the following Table 1.

The original LPV is a function of SBH, which is closely related to metal work function. Therefore, Table 1 reveals a law that there is a negative correlation between the modification range of SBH through charge trapping effect and the original SBH, or the work function of metal material. This rule can be simply attributed the tolerance capacity of oxide layer to built-in electric field (Schottky barrier). Obviously, the oxide layer can’t always hold a difference of Fermi level no matter how strong the built-in field is. The higher the field is, the easier for electrons to tunnel through oxide layer. Therefore, low work function materials show better control characteristics than high work function materials.

In summary, we discovered a nonvolatile and tunable switching effect of lateral photo-voltage in metal dusted metal-oxide-semiconductor structure. By combining the application of a short voltage pulse with laser illumination, the sensitivity and linearity of lateral photo-voltage can be adjusted in a nonvolatile manner. Based on a photo generated electrons trapping model, the variation of Schottky barrier height and film resistivity was proposed to account for this phenomenon. Compared to common LPV devices, the tunable and nonvolatile features of this effect may endow it a wider application areas.

## Additional Information

**How to cite this article**: Zhou, P. *et al.* Nonvolatile and tunable switching of lateral photo-voltage triggered by laser and electric pulse in metal dusted metal-oxide-semiconductor structures. *Sci. Rep.* **6**, 32015; doi: 10.1038/srep32015 (2016).

## Figures and Tables

**Table 1:** The performance of different materials with same thickness under same treatment of +20 V pulse & laser.

Material	Work function	Original LPV	Final LPV	LPV increment	Incremental ratio
n-Si	4.31	—	—	—	—
Ti	4.33	11.2 mV	78.3 mV	67.1 mV	599%
Cu	4.94	41.6 mV	121.7 mV	80.1 mV	193%
Pt	5.84	76.3 mV	91.8 mV	15.5 mV	20.3%
